# Influence of Strength Training Variables on Neuromuscular and Morphological Adaptations in Prepubertal Children: A Systematic Review

**DOI:** 10.3390/ijerph20064833

**Published:** 2023-03-09

**Authors:** Alberto Sánchez Pastor, Carlos García-Sánchez, Moisés Marquina Nieto, Alfonso de la Rubia

**Affiliations:** 1 Facultad de Ciencias de la Actividad Física y del Deporte, Universidad Politécnica de Madrid, Calle Martín Fierro 7, 28040 Madrid, Spain; 2 Deporte y Entrenamiento Research Group, Departamento de Deportes, Facultad de Ciencias de la Actividad Física y del Deporte, Universidad Politécnica de Madrid, Calle Martín Fierro 7, 28040 Madrid, Spain

**Keywords:** resistance, power, training, prepubescent, youth, adaptations, performance, non-experience

## Abstract

Strength training in prepubertal children is one of the topics that has aroused the most interest and controversy among training professionals in recent years. Therefore, the aim of the present study was to analyze the available scientific evidence on the influence of strength training variables on morphological and/or neuromuscular adaptations in healthy prepubertal populations with no previous experience in this type of training according to the descriptive sample characteristics. According to the Preferred Reporting Items for Systematic Reviews and Meta-Analysis, 22 studies were selected after a systematic search and selection process using four electronic databases: Google Scholar, PubMed, Scopus, and SPORT Discus. Furthermore, the internal validity of the studies included was assessed using the modified PEDro scale. The sample consisted of 604 prepubertal children (age, 10.02 ± 0.75 years), of whom 473 were boys and 131 were girls, with 104 strength training programs recorded. Strength training resulted in a significant increase in jumping (*n* = 29) and sprinting (*n* = 13) abilities. Moreover, muscle strength was increased in 100% of the cases. Morphologically, strength training resulted in a decrease in body fat percentage (*n* = 19) and an increase in lean body mass (*n* = 17). With regard to gender, increases in general sport skills and basic physical abilities were significant in males but not in females. Thus, the results are more heterogeneous in girls due to the small number of studies carried out. Therefore, this research provides practical applications for coaches to design and implement more effective training programs to maximize adaptations, enhance physical performance, and reduce injury risk.

## 1. Introduction

Muscle strength is the ability to exert a force on an external object or resistance [1,2]. Therefore, strength or resistance training can be defined as a method to develop musculoskeletal fitness through a broad spectrum of loads with different purposes, such as increasing sport performance [3], improving health [4], and preventing and rehabilitating injuries [5]. As a consequence of this kind of training, adaptations are produced at different levels (e.g., neuromuscular) through the manipulation of strength training variables (e.g., duration) [6], which must be adjusted to a series of biological and psychological demands specific to each individual [7]. The scientific literature has demonstrated the effects of strength training in population samples of different characteristics such as elderly people or nonagenarians [8], older adults [9], sedentary people [10], obese subjects [11], pregnant women [12], and athletes [13].

One of the populations in which strength training has attracted the most interest in recent years is prepubertal children [14,15]. However, strength training in prepuberty has been a controversial subject, and there has been a great diversity of opinions among different health and sports professionals. In the first studies published during the 1970s and 1980s [16,17], no beneficial effects were found, leading to generalized conclusions based on its ineffectiveness in children and/or adolescents [18]. Nowadays, and from the clear position statements of the National Strength and Conditioning Association in its latest updates [19,20,21], it can be affirmed that strength training in prepuberty supervised by qualified adult professionals who use simple and understandable language and whose focus is on the appropriate development of the execution technique is effective and safe in terms of health and performance. Furthermore, the American College of Sports Medicine (ACSM) establishes as a priority objective for strength training at early ages the improvement of musculoskeletal strength and overall fitness level through exposure to a variety of safe, effective, and enjoyable training methods. Specifically, the ACSM suggests performing three sets of 6–15 repetitions on two non-consecutive days per week using a variety of equipment that has been shown to be safe (i.e., medicine balls, free weights, or machines) [22]. Along the same lines are the recommendations of the World Health Organization (WHO) [23] suggesting 60 min of moderate to intense physical activity incorporating muscle and bone strengthening activities 3 days a week, which can be sports or recreational activities. Based on this international consensus and an amalgam of scientific publications, it has been possible to demystify certain risks or disadvantages of strength training in prepubertal children. In this development stage—up to the age of 11–13 years in girls and boys, respectively [24], corresponding to stage 1 of the Tanner scale [15]—one of the main arguments against strength training has been the detrimental effects on body and bone growth. A number of studies [25,26] proposed delaying the age at which strength training should begin, arguing that insufficient hormone levels in bone structures would not allow them to withstand overloads and, therefore, could lead to alterations in the ossification process or deformities. However, there is no scientific evidence in this regard; on the contrary, various retrospective [19] and prospective studies [27] concluded that subjecting growth plates to mechanical stress through strength training was beneficial for body and bone growth. A powerful stimulus on bone structures can be obtained through strength training based on moderate- to high-intensity multi-joint exercises and the introduction of plyometric exercises [19]. Another of the most frequently cited factors for the contraindication of strength training in prepuberty was high injury incidence and risk. However, the scientific literature shows that strength training at an early age helps to reduce overuse injuries by 50% [28]. Specifically, in young athletes, supervised global strength training leads to an increase in physical conditioning levels that allows them to face the musculoskeletal demands derived from physical activity and sport with guarantees, resulting in a lower injury incidence [19,20,21,29]. Moreover, strength training not only helps to reduce injury risk but is also used in injury prevention [30] and rehabilitation work [31].

The adaptations derived from strength training in prepuberty through the manipulation of strength training variables (i.e., volume) tend to respond to a greater extent to physiological neural mechanisms and to a lesser extent to morphological mechanisms [32]. On the one hand, the main neuromuscular adaptations produced by strength training are motor unit recruitment, firing frequency, and synchronization and intermuscular coordination [33], which have been shown to be the fundamental factors of performance improvements in general basic skills such as throwing, running, and jumping [34]. On the other hand, there are morphological adaptations which are not so frequent and determinant at early ages [35], and which occur not only in muscles (i.e., increase in cross-sectional area, fascicle length, and pennation angle) [36] but also in other tissues such as bone, preserving bone mineral density [37] and improving tendon stiffness [38].

Some researchers [20,39] have recommended that an effective strength training program for adolescents (12–18 years in girls and 14–18 years in boys) should have the following characteristics: process duration between 8 and 12 weeks, training frequency of 2.7 ± 0.8 sessions per week, volume of 3 to 8 exercises and 1–2 sets per exercise, intensity between 60 and 85% of 1 RM, moderate velocities focusing on controlled exercise execution, and intervals lasting between 1 and 3 min. Furthermore, with regard to the nature of the exercises, Lloyd et al. [20] proposed a gradual progression from simple and stable exercises to complex and unstable exercises with the aim of allowing a distributed and balanced involvement of the main muscle groups of the whole body. However, it should be noted that an optimal combination of strength training variables has not been established for strength training in prepubertal children. Therefore, these general guidelines for adolescents should be adapted with caution depending on the prepubertal children’s experience level in relation to the physical activity level and the training process [40].

To the best of our knowledge, this systematic review is one of the first studies to synthesize and evaluate the current scientific knowledge with regard to the prescription of strength training in prepubertal children. Therefore, the aim of the present study was to analyze the available scientific evidence on the influence of strength training variables on morphological and/or neuromuscular adaptations in healthy prepubertal populations according to the descriptive sample characteristics.

## 2. Materials and Methods

The stages of the procedure employed for the present systematic review adhered to both the Preferred Reporting Items for Systematic Reviews and Meta-Analysis (PRISMA) checklist and the Population, Interventions, Comparisons, Outcomes, and Study Design (PICOS) question model for the definition of inclusion criteria [41], as well as the PROSPERO guidelines (registration no. CRD42022360557).

### 2.1. Study Eligibility Criteria: Inclusion and Exclusion

Original scientific research based on strength training in prepubertal children was considered. Studies were published in peer-reviewed journals with an impact factor included in the Journal Citation Reports of the Web of Science (JCR of WoS).

According to the Population, Interventions, Comparisons, Outcomes, and Study Design (PICOS) question model [42], the inclusion criteria were as follows: (1) Population—prepubertal children under 12–13 years [43] with no previous experience in strength training; (2) Intervention—application of a strength training program in a prepubertal population with homogeneous characteristics; (3) Comparison—evaluation between the results of the experimental group and the results of the control group and/or with other training methods; (4) Results—morphological and/or neuromuscular adaptations (increases and decreases, whether significant or not) considering the strength training variables and according to the descriptive characteristics of the sample; (5) Study Design—retrospective descriptive-observational study.

The exclusion criteria were as follows: (1) adolescent or adult subjects; (2) prepubertal children with some type of pathology or health problem that could condition the practice of physical activity (e.g., obesity); (3) training programs focused on a physical capacity other than strength or combined training (i.e., concurrent training); (4) application of strength testing or assessment as complementary content within the sports talent development process (e.g., football, basketball, or volleyball); (5) strength training aimed at rehabilitation or readaptation; (6) failure to provide comprehensive information about strength training methods. Systematic reviews and other types of articles (i.e., conferences or editorials) associated with the study objective were not included for formatting reasons.

### 2.2. Search Strategy and Systematic Review Protocol

The search process for published scientific studies based on strength training in prepubertal children was carried out through four electronic databases (Google Scholar, PubMed, Scopus, and SPORT Discus) and by reviewing email alerts from research databases, with no restriction on publication date. The keywords that formed the two search strings were (1) ‘child’ OR ‘children’ OR ‘kid*’ OR ‘preadolescent*’ OR ‘prepubertal’ OR ‘middle childhood’ OR ‘infant*’ OR ‘early childhood’ AND (2) ‘strength training’ OR ‘resistance training’ OR ‘strength program*’ OR ‘fitness’ OR ‘weight training’ OR ‘elastic band training’ OR ‘free weight training’ OR ‘maximum strength’ OR ‘strength workout’ OR ‘plyometric’.

**Figure 1:**
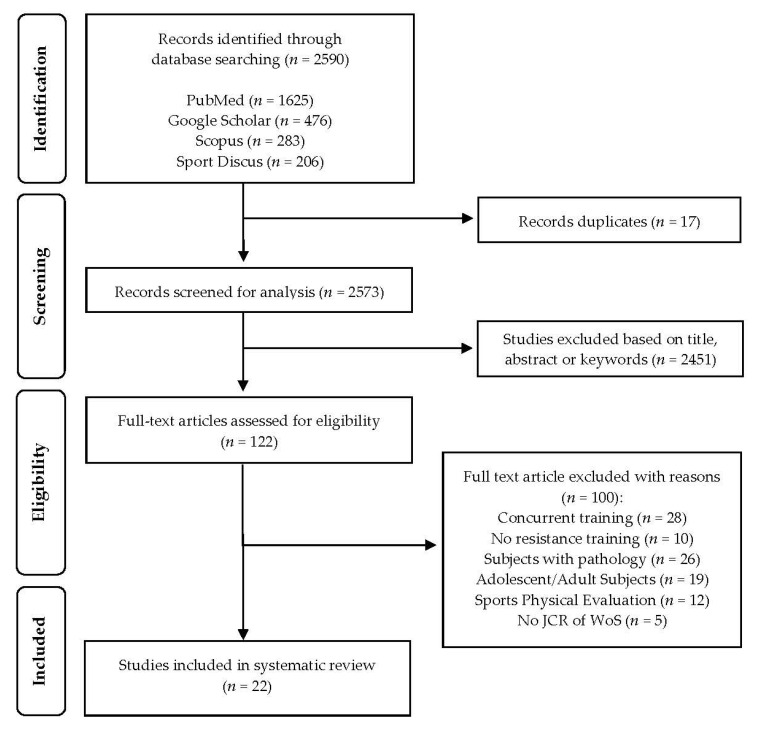
Flow diagram for screening and selection of studies according to Preferred Reporting Items for Systematic Reviews and Meta-Analysis (PRISMA).

According to the criteria for preparing systematic reviews—PRISMA [41]—the protocol was carried out in the months of October and December 2022 and was composed of four stages (Figure 1). (1) Identification: The first (A.S.P.) and fourth (A.R.R.) authors found a total of 2590 studies in the four databases consulted (Google Scholar, *n* = 476; PubMed, *n* = 1625; Scopus, *n* = 283; SPORT Discus, *n* = 206). (2) Screening: After the elimination of duplicate records (*n* = 17) by the first author (A.S.P.), 2573 articles were considered for further analysis. (3) Eligibility: After reading the title, abstract, and/or keywords, the first (A.S.P.) and second (C.G.-S.) authors ruled out 2451 articles, leaving 122 records at the end of this phase. (4) Inclusion: After complete reading, 99 articles were excluded by the first (A.S.P.), second (C.G.-S.), third (M.M.N.), and fourth (A.R.R.) authors for the reasons of adolescent age (*n* = 19), children with pathology or disease (*n* = 26), concurrent training (*n* = 28), not performing a strength training protocol (*n* = 10), physical sports tests or assessments (*n* = 12), and not being included in the JCR of WoS (*n* = 4). Finally, the total number of studies included in the systematic review was 22. The authors worked separately and independently to ensure the reliability of the process and the eligibility of the studies.

### 2.3. Data Extraction

A standardized form was used to extract data from the studies included in the review for assessment of the study quality and scientific evidence. Thus, the following information was collected: (A) year of publication; (B) author/s; (C) sample characteristics, including (C1) number of prepubertal children (*n*), (C2) gender (men and women), (C3) age, and (C4) physical activity level (PAL; tier 0 or sedentary children (SdCh), tier 1 or recreationally active children (RcCh), and tier 2 or trained children (TrCh)); (D) strength training program (STP) and strength training variables (STVs), including (D1) periodization, (D2) duration, (D3) frequency, (D4) intensity, (D5) volume, (D6) movement velocity, (D7) rest interval, (D8) joints involved, and (D9) exercise type; (E) morphological adaptations (MAs), including (E1) body composition—body fat (BC-BF) and (E2) body composition—lean body mass (BC-LBM); (F) neuromuscular adaptations—general sport skills (NA-GSS), including (F1) jump and (F2) sprint; (G) neuromuscular adaptations—basic physical abilities (NA-BPA), including (G1) strength, (G2) agility, (G3) coordination, (G4) balance, and (G5) flexibility. Moreover, the number of STPs that resulted in a significant or non-significant increase or decrease in the abovementioned STVs was recorded. Table 1 presents the references used for the design and coding of the variables and categories corresponding to the sample and strength training programs’ characteristics.

### 2.4. Study Quality Assessment

The internal validity of the studies included in this systematic review was assessed using the modified ‘Physiotherapy Evidence Database—PEDro scale’ [54]. This scale consists of 11 items, of which one refers to the external quality of the studies (item 1) while the remaining ten refer to the internal quality (items 2–11). If the item was satisfied in the study assessed (‘Yes’), one point was awarded, and if it was not satisfied (‘No’), zero points were awarded. Therefore, the rating of the studies ranged from a minimum score of 0 points to a maximum of 10 points. The total score was interpreted as follows according to the methodological quality: excellent quality (9–10 points), good quality (6–8 points), acceptable quality (4–5 points), and poor quality (0–3 points). The study quality assessment was carried out by two independent reviewers (A.S.P. and A.R.R.). The second (C.G.-S.) and third (M.M.N.) authors resolved disagreements in the rating, and inter-rater reliability was calculated.

## 3. Results

### 3.1. Study Characteristics—Sample

Table 2 shows, in chronological order, the sample characteristics of the scientific evidence included in the present systematic review. In the 22 studies, 604 prepubertal children (473 boys and 131 girls; age, 10.02 ± 0.75) were registered, all of whom were classified as Tanner stage I [55]. With regard to PAL, in six studies, prepubertal children engaged in occasional/incidental physical activity (28.15%)—tier 0 or sedentary (SdCh); in seven studies, subjects belonging to tier 1 were engaged in recreational physical activity (RcCh) several times a week (40.23%); and in eight studies, prepubertal children belonging to tier 2 trained with the aim to compete in a specific sport (TrCh) on a regular basis (27.65%). One study [56] did not present the PAL of the subjects (3.97%).

### 3.2. Strength Training Variables

The number of STPs implemented in the prepubertal population was 104 (Table 3). In relation to the different STVs identified, the main characteristics of the STPs were linear/non-linear periodization (83.65%), duration between 8 and 12 weeks (81.73%), frequency of two days per week (81.73%), moderate intensity (61.76%), low volume (88.46%), high movement velocity (82.86%), rest intervals between 1 and 2 min (51.02%), composed of multi-joint exercises (72.12%), and machine-based exercises as the main exercise type (36.54%). It is worth noting the lack of information in the literature analyzed regarding some strength training variables, such as intensity (*n* = 2), movement velocity (*n* = 69), and rest intervals (*n* = 55).

### 3.3. Strength Training Variables—Main Adaptations

Table 4 shows the main neuromuscular (NAs) and morphological (MAs) adaptations from the STPs according to the strength training variables (STVs). With regard to neuromuscular adaptations, for general sport skills (NA-GSS), significant increases in jumping ability were identified after 29 STPs, with no decreases recorded. In relation to sprinting ability, 13 STPs resulted in a significant increase. By contrast, two STPs had a negative impact on sprint performance. As for basic sport abilities (NA-BPA), all STPs had positive effects (66 significant increases and 28 increases) on muscular strength, without decreases. Agility and coordination abilities also showed significant increases post-STP (*n* = 5 and *n* = 2, respectively). On the other hand, increases were detected with regard to flexibility (*n* = 16) and balance (*n* = 6). Considering morphological adaptations (MAs), specifically body composition—body fat (BC-BF), 19 STPs resulted in a decrease in body fat percentage while 2 STPs were recorded as having led to an increase. Regarding lean body mass (LBM), 17 STPs resulted in an increase in lean mass while 4 STPs resulted in a decrease.

**Figure 2:**
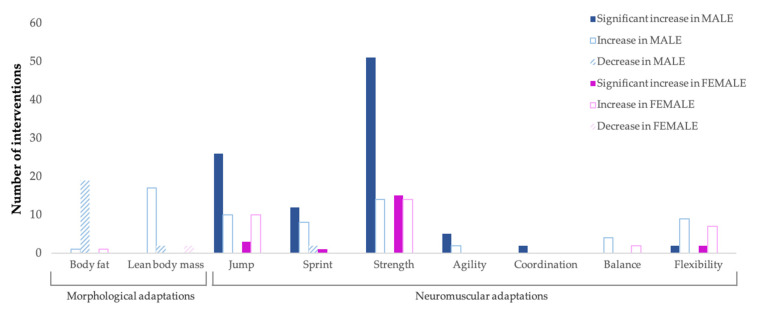
Gender-specific neuromuscular and morphological adaptations from STPs.

**Figure 3:**
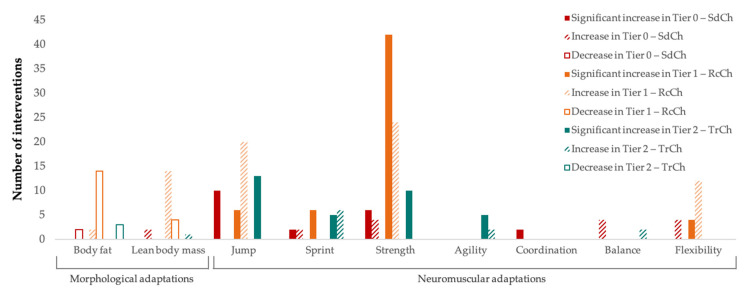
Neuromuscular and morphological adaptations from STPs according to PAL.

Figure 2 and Figure 3 show the adaptations from the STPs according to gender and PAL, respectively. With regard to morphological adaptations, the scientific evidence focuses almost exclusively on boys (39 out of 42 interventions), with decreases in BF in 95% of cases and increases in LBM in 89.47%. In girls, only three interventions were performed with similar results. In relation to neuromuscular adaptations, significant increases in jumping ability (*n* = 26), sprinting ability (*n* = 12), muscle strength (*n* = 51), agility (*n* = 5), coordination (*n* = 2), and flexibility (*n* = 2) were recorded throughout the 147 STPs implemented in boys. Meanwhile, in girls, most of the increases detected (jumping, *n* = 10; balance, *n* = 2; and flexibility, *n* = 7) in the 54 STPs developed were not significant. With regard to PAL, the most examined population group was RcCh (tier 1) (*n* = 34), with decreases in BF and increases in LBM in 87.50% of the cases. In relation to neuromuscular adaptations, the 114 STPs implemented on SdCh (tier 0), RcCh (tier 1), and TrCh (tier 2) produced significant increases in muscle strength (*n* = 6, *n* = 42, and *n* = 10, respectively), jumping (*n* = 10, *n* = 6, and *n* = 13, respectively), and sprinting abilities (*n* = 2, *n* = 6, and *n* = 5, respectively).

### 3.4. Influence of Strength Training Variables on Muscular Strength

**Figure 4:**
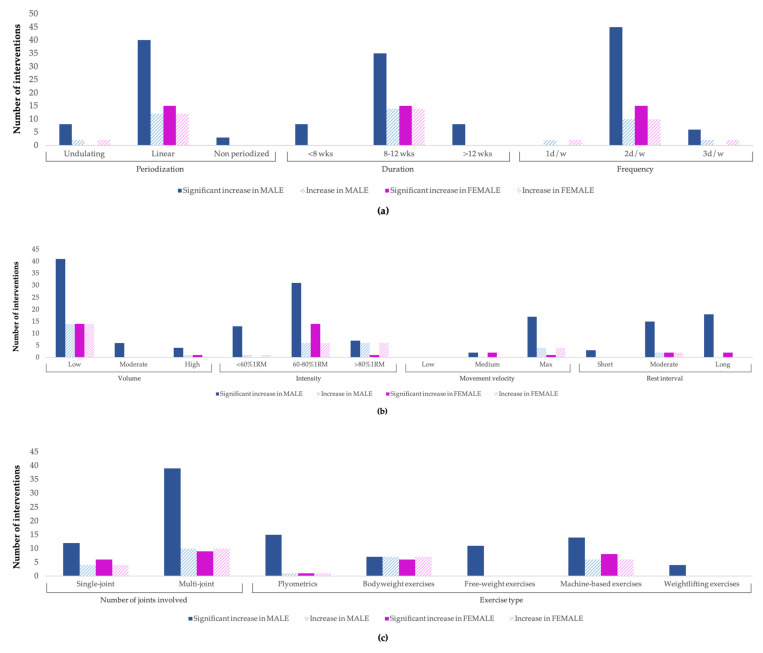
(**a**–**c**) Three-block adaptation effects of STVs on muscle strength according to gender.

Figure 4a (periodization, duration, and frequency), Figure 4b (volume, intensity, movement velocity, and rest interval), and Figure 4c (joints involved and exercise type) report the adaptation effects of each of the STVs on muscle strength according to gender. The record of the studies analyzed shows clear results in boys (e.g., four times more significant increases in muscle strength from STPs with a duration of 8–12 weeks (*n* = 35) than in interventions of less than 8 weeks (*n* = 8) or more than 12 weeks (*n* = 8)), while in girls, the conclusions do not seem so clear.

### 3.5. Influence of Strength Training Variables on Jumping and Sprinting Abilities

**Figure 5:**
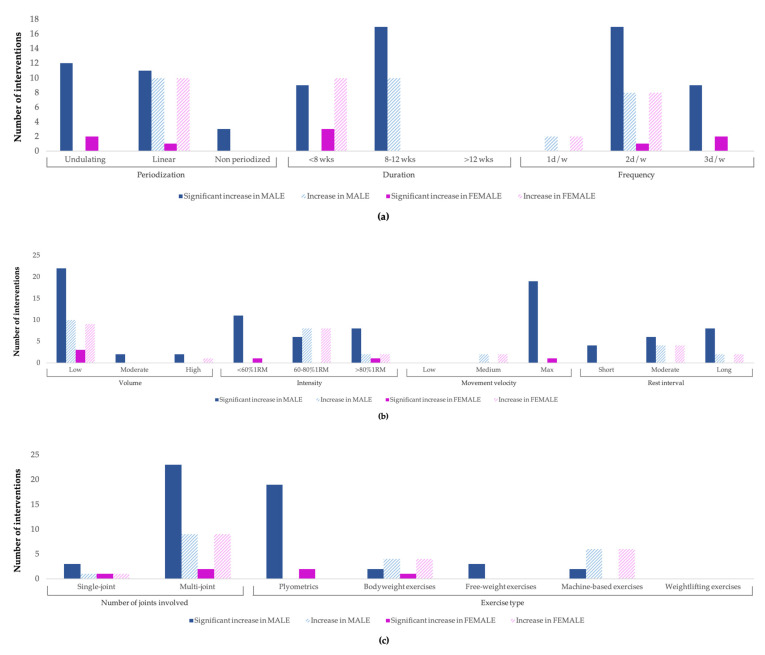
(**a**–**c**) Three-block adaptation effects of STVs on jumping ability according to gender.

Figure 5a (periodization, duration, and frequency), Figure 5b (volume, intensity, movement velocity, and rest interval), and Figure 5c (joints involved and exercise type) report the adaptation effects of each STV on jumping ability according to gender. The higher number of interventions in boys showed more significant increases in jumping ability in STPs with the following characteristics: between 8 and 12 weeks (*n* = 17), 2 days/week (*n* = 17), low volume (*n* = 22), maximal movement velocity (*n* = 19), multi-joint (*n* = 23), and plyometric (*n* = 19) exercises. Other STVs did not demonstrate differences between their categories (i.e., periodization, intensity, and rest interval). In girls, significant increases were observed in STPs with a duration of less than 8 weeks (*n* = 3) and frequency of 2 or 3 days per week (*n* = 1 and *n* = 2, respectively), with no interventions in relation to rest interval.

**Figure 6:**
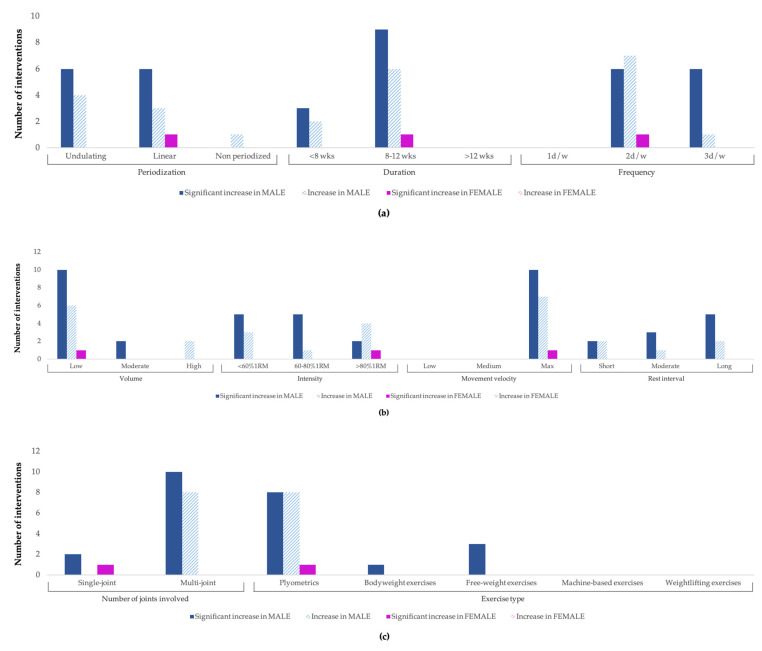
(**a**–**c**) Three-block adaptation effects of STVs on sprinting ability according to gender.

Figure 6a (periodization, duration, and frequency), Figure 6b (volume, intensity, movement velocity, and rest interval), and Figure 6c (joints involved and exercise type) report the adaptation effects of each of the STVs on sprinting ability according to gender. The results in boys are similar to those observed for jumping ability, except in relation to training frequency, where significant increases in sprinting ability were found for both 2 days per week (*n* = 6) and 3 days per week (*n* = 6). The lack of studies in girls yields no conclusions associated with sprinting ability according to STVs.

### 3.6. Study Selection and Assessment (Quality Analysis)

The quality analysis using the PEDro scale yielded the following results (Table 5). The quality scores ranged from 4 to 7, with an average score of 6.05 points. Additionally, of the twenty-two included studies, one (4.55%) was considered to be of “acceptable quality” (4–5 points), and the remaining twenty-one studies (95.45%) were considered to be of “good quality” (6–8 points). No studies were categorized as “poor quality” or “excellent quality”. The highest scores by criteria (one point) were located in items 2, 9, 10, and 11 (100%), followed by items 4 (90.91%) and 8 (95.45%). On the other hand, the most commonly unsatisfied items (zero points) were items 5, 6, and 7 (100%).

## 4. Discussion

The present study represents the most comprehensive and exhaustive systematic review to analyze the available scientific evidence on morphological and/or neuromuscular adaptations derived from the application of strength training programs in healthy prepubertal populations. The main strengths of this research lie in the qualities of the external validity and generalizability of the results from the analysis of several strength training programs (*n* = 104) and the large sample of prepubertal children (*n* = 604). However, the final sample was composed of a lower number of girls (*n* = 131) compared with boys (*n* = 473). Additionally, a lower number of interventions provided information about morphological adaptations (*n* = 19) compared with neuromuscular adaptations (*n* = 85). Moreover, nine studies provided information about morphological and neuromuscular adaptations.

### 4.1. Neuromuscular Adaptations

The studied STPs had a positive impact on neuromuscular adaptations (GSS and BPT) in healthy prepubertal children. Specifically, the STPs produced significant increases, especially in boys, in jumping ability, sprinting ability, muscle strength, agility, coordination, and flexibility. In relation to PAL, the STPs produced significant increases in jumping ability, sprinting ability, and muscle strength in all groups. Moreover, the STPs produced significant increases in coordination in the SdCh group (tier 0), flexibility in the RcCh group (tier 1), and agility in the TrCh group (tier 2). These findings confirm that strength training provides many benefits in prepubertal children, as in other populations [8,9,10,11,12,13]. Furthermore, regarding the strength training variables, the greatest neuromuscular adaptions occurred in programs with a duration of 8–12 weeks, a frequency of 2 days/week, low volume, moderate intensity, high movement velocity, medium or long rest intervals, multi-joint exercises, and machine-based or plyometrics exercises. These results are in line with previous research recommendations [20,21].

The findings in relation to the load organization and sequencing show that linear periodization was the most commonly used in STPs that improved muscular strength. Similar results were found in previous research conducted with untrained subjects [78,79]. On the other hand, both linear and undulating periodization had positive effects on sprinting and jumping in boys and girls. As the subjects were inexperienced in strength training, it is likely that the periodization type employed did not play a determinant role in the adaptations produced [45]. However, as the subject’s experience increases, undulating periodization may become more effective in increasing muscle strength and avoiding possible injuries associated with overtraining [9,45,50].

With regard to the strength training quantity, most of the interventions that improved muscle strength in both boys and girls lasted between 8 and 12 weeks. With the same duration (8–12 weeks), increases in jumping and sprinting abilities were recorded in boys. However, an increase in jumping ability in girls was identified in STPs of less than 8 weeks. Numerous studies indicate that between 8 and 12 weeks, muscle strength increases are mainly produced by neural adaptations, especially due to the high neural plasticity and rapid myelination changes that occur in prepuberty [32,33,34,36,80]. However, Vingren et al. [81] pointed out that small morphological adaptations also occur during this time interval, although they are probably not significant for strength gain due to the lack of androgenic hormone production. In relation to frequency, the STPs that led to significant improvements in muscular strength and jumping and sprinting abilities in boys and girls took place two days a week. Along the same lines, previous research recommended training two or three days a week on non-consecutive days to allow children sufficient recovery time between sessions [20,21]. Indeed, de Villareal et al. [82] demonstrated that moderate or low frequencies of plyometric training produced greater improvements in jumping and sprinting abilities. Nevertheless, there is some controversy regarding the use of a low training frequency (1 day/week). While some studies indicated that this frequency may not be sufficient to improve muscular strength [27], others stated that it may be sufficient to maintain or improve muscular strength levels in untrained children [9,19]. In addition, significant improvements in muscle strength and jumping and sprinting abilities corresponded to low training volumes in both boys and girls. These findings are consistent with recommendations developed for children with little or no training experience [20,51]. Furthermore, some studies showed that a higher training volume (moderate or high) did not produce significant improvements in muscle strength in trained subjects because it could generate an excessive endocrine response, leading to high fatigue levels [83,84]. Therefore, at the beginning of an STP, it would be recommended that children perform a low number of repetitions (1–6) and sets (1–2), and they should receive constant feedback (internal and external cues) after each repetition in order to develop a proper execution technique [20,21,51].

In terms of the strength training quality, significant muscle strength gains in both boys and girls were recorded in STPs at moderate intensities (60–80% RM). However, this finding should be considered with caution, as the intensity must be adapted to children’s technical ability and muscular strength levels [20]. Furthermore, it should be considered that a training session performed at 80% RM does not involve the same effort degree and difficulty as a training session performed at 60% RM [7], both being classified as moderate intensity. By contrast, lower intensities corresponded to significant improvements in sprinting and jumping abilities, especially in children. Several investigations (i.e., Baena-Marín et al. [85]) pointed out that low-intensity training performed at maximal movement velocity during the concentric phase would increase the athlete’s ability to apply force in a short time, as occurs in jumping and sprinting actions. In line with the above argument, significant increases in muscle strength and sprinting and jumping abilities were identified in STPs carried out at high or maximal movement velocities in both boys and girls. Performing strength exercises at maximal intended velocity allows for greater gains in muscle strength due to the optimization of neuromuscular performance in explosive actions such as jumping and sprinting [50,71,86,87]. However, some studies recommend that when starting an STP with untrained subjects, the movement velocity should be moderate and controlled in order to acquire a proper execution technique and reduce the injury risk [64]. Rest between exercises is another fundamental factor in the strength training quality. Notably, significant increases in muscle strength and sprinting and jumping abilities were observed in STPs that used moderate or long rest intervals. These results could be explained by the study from Schoenfeld et al. [88], in which it was found that long rest periods favor muscular strength gains, especially in the lower limbs due to the increase in thickness in a muscle that is so decisive in jumping and sprinting actions such as the quadriceps femoris. However, in relation to sprint actions, short or moderate rest intervals may be sufficient [52]. In prepubertal children, this may be due to their greater recovery capacity, produced by their limited power generation capacity due to their greater difficulty in recruiting higher hierarchical motor units and lower peak blood metabolites [89]. Despite this, practitioners should monitor the rest intervals to guarantee that correct exercise technique is maintained during the entire session [20,51].

Associated with the exercise selection, the findings of the present systematic review reveal significant increases in muscle strength and jumping ability with multi-joint exercises, as well as in sprinting ability in boys only. The involvement of several joints in exercises causes greater muscle mass mobilization [36] and requires a greater capacity for intermuscular coordination during the movement, resulting in a reduction in the coactivation of the antagonist musculature [32]. Likewise, multi-joint exercises involving the lower limbs generate a greater transfer to actions such as jumping and sprinting due to a greater specificity of training [90]. With regard to exercise type, a greater gain in muscle strength was identified in exercises with machines and with own body weight. In boys, significant improvements were also identified with the use of plyometric exercises. However, few studies have used free weight or weightlifting exercises in their intervention programs, probably because these require a high technical skill level and a longer familiarization period [32]. This could be the reason why machine or bodyweight exercises are chosen for prepubertal children, because they are easier and quicker to learn [40]. Furthermore, plyometric exercises were the most used to improve jumping and sprinting abilities. Based on the development of the stretch–shortening cycle (SSC) and focusing on using elastic energy and reflexive muscle activity mechanisms [50], these exercises produce tendon adaptations (e.g., increased stiffness), being determinant to increase performance in explosive actions such as jumping and sprinting [82,91].

### 4.2. Morphological Adaptations

The STPs had a clearly positive impact in morphological adaptations (BF and LBM), regardless of gender and PAL. However, the scientific evidence focuses almost exclusively on boys (39 out of 42 interventions), with decreases in BF in 95% of cases and increases in LBM in 89.47%. With regard to PAL, the most examined population group was RcCh (tier 1), with decreases in BF and increases in LBM in 87.50% of the cases. Therefore, although the results showed positive adaptations in LBM, the scientific evidence indicates that muscle mass gain in prepubertal children is very low or non-existent [27,39]. Thus, this gain is probably due to the muscle tissue increase caused by the children’s maturational development [92]. On the other hand, the BF decrease was probably due to the increased energy expenditure resulting from the strength training, causing, as a consequence, a caloric deficit [93,94]. However, in two interventions, the BF increased. This result could be explained by the length of the study—28 weeks—because during that time, the main factor responsible for the BF increase would be the prepubertal maturational development process itself [95,96].

### 4.3. Limitations

This systematic review has the following limitations: (i) a low number of STPs had a duration longer than 12 weeks; (ii) there was a low number of female participants; (iii) few studies measured morphological changes in muscle, tendon, and bone; (iv) no studies reported hormonal measures (e.g., testosterone); (v) some STPs did not provide information about some strength training variables, such as intensity, movement velocity, and rest intervals; (vi) some studies did not report changes according to gender.

### 4.4. Practical Applications

Considering the main findings of the selected studies, some practical recommendations for designing and implementing STPs in prepubertal children are presented: (i) Ensure the use of a duration equal to or greater than 8 weeks. (ii) Adapt the strength training variables to the children’s training level. At the beginning, it is advisable to start with low frequencies, volumes, intensities, and movement velocity (focused on technical proficiency). Subsequently, progress to moderate frequencies, volumes, and intensities and high or maximum movement velocity. (iii) Prescribe short or moderate rest intervals to minimize the impact of fatigue on the technical skill of exercises. (iv) Prioritize the use of multi-joint exercises due to the involvement of a greater muscle mass, having a positive transfer to jumping and sprinting abilities. (v) Employ machine-based exercises, bodyweight exercises, and plyometrics in the early phases of strength training. After consolidating the basic movement patterns, such as squatting, hip hinging, pushing, pulling, jumping, landing, and hopping, it would be recommended to progressively introduce free weights or weightlifting exercises. (vi) Consider strength training as a tool to prevent overweight and obesity. Our recommendations are in line with the ACSM and WHO guidelines for prepubertal children.

### 4.5. Future Research Lines

Further studies will be needed to deepen our knowledge in the following topics: (i) the influence of other strength training variables such as range of motion, repetitions in reserve, exercise order, passive or active rest, and type of muscle contraction; (ii) the morphological adaptations produced by strength training to other structures such as muscle (e.g., cross-sectional area, pennation angle, and fascicle length), tendons (e.g., stiffness), ligament (e.g., thickness), and bone (e.g., density); (iii) the adherence generated by game-based strength training programs vs. traditional strength programs; (iv) the adaptations from strength training in subjects under 7 years; (v) the injury incidence caused by strength training in prepubertal children.

## 5. Conclusions

This systematic review confirms the efficacy of strength training to increase neuromuscular and morphological adaptations in prepubertal children. In relation to neuromuscular adaptations, strength training produces significant increases in jumping and sprinting abilities, muscle strength, agility, coordination, and flexibility, especially in boys. With regard to morphological adaptations, strength training generates significant increases in lean body mass and significant decreases in body fat, regardless of gender and physical activity level. Therefore, this research provides practical applications for coaches and practitioners to design and implement more effective training programs to maximize adaptations (morphological and neuromuscular), enhance physical performance, and reduce injury risk according to gender and physical activity level.

## Figures and Tables

**Table 1:** Study strength training variables (STVs) categorized according to scientific evidence in relation to sample characteristics (SCs) and strength training program (STP).

STV	Categories	Reference
Physical Activity Level (SC)	Tier 0—Sedentary children (SdCh)	Extracted from McKay et al. [44]
	Tier 1—Recreationally active children (RcCh)	
	Tier 2—Trained children (TrCh)	
Periodization (STP)	Undulating periodization	Extracted from Williams et al. [45]
	Linear/non-linear periodization	
	No periodization	
Duration (STP)	<8 weeks	Extracted from Clark et al. [46] and Lesinski et al. [47]
	8–12 weeks	
	>12 weeks	
Frequency (STP)	1 day/week	Extracted from Faigenbaum and Myer [40] and Grgic [48]
	2 days/week	
	3 days/week	
Intensity (STP)	Low: TSE → <60% 1 RM Ply → Jumps in place and standing jumps	TSE—extracted from Cormie et al. [33] Ply—modified from Williams [49] TSE and Ply—adapted from Fort-Vanmeerhaeghe et al. [50]
	Moderate: TSE → 60–80% 1 RM Ply → Bounding and hurdles	
	High: TSE → >80% 1 RM Ply → Depth jumps and drop jumps	
Volume * (STP)	Low (18–278 set/reps/exercise)	Adapted from Peña et al. [39] and Ralston et al. [51]
	Moderate (279–540 set/reps/exercise)	
	High (541–800 set/reps/exercise)	
Movement Velocity (STP)	Low	Extracted from Lloyd et al. [20]
	Moderate	
	High	
Rest Interval (STP)	Short (0–1 min)	Extracted from Grgic et al. [52] and Peña et al. [39]
	Medium (1–2 min)	
	Long (+2 min)	
Joints Involved (STP)	Single-joint	Extracted from Peña et al. [39] and Suchomel et al. [53]
	Multi-joint	
Exercise Type (STP)	Plyometrics (unilateral/bilateral)	Extracted from Suchomel et al. [53]
	Bodyweight exercises	
	Free weight exercises	
	Machine-based exercises	
	Weightlifting exercises	

Notes: TSE, traditional strength exercises; Ply, plyometrics; 1 RM, one repetition maximum. * According to the minimum and maximum volume registered in the sample studies.

**Table 2:** Summary of sample characteristics (author, year, gender, age, and physical activity level (PAL)) and aim(s) of the studies.

Author(s)	Year	Gender (*n*)		Age (X ± SD)	PAL	Aim(s) of the Study
		M	F			
Tsolakis et al. [57]	2000	9	-	11.78 ± 0.84	Tier 0—SdCh Sedentary Lifestyle	To investigate the influence of a short (2-month), supervised, progressive resistance training program with isotonic equipment and a 2-month detraining program on T, sex hormone-binding globulin (SHBG), and free androgen index (FAI) blood concentration in two different age groups of untrained prepubertal and pubertal Greek boys
Diallo et al. [58]	2001	10	-	12.30 ± 0.40	Tier 2—TrCh Regularly Training	To determine the effects of short-term plyometric training and detraining on performance in pubescent soccer players
Faigenbaum et al. [59]	2001	44	22	8.10 ± 1.60	Tier 1—RcCh Recreational Physical Activity	To examine the effects of four different resistance training protocols on upper body performance adaptations in healthy children
Fuchs et al. [60]	2001	25	20	7.50 ± 0.16	Tier 1—RcCh Recreational Physical Activity	To examine the effects of a high-intensity jumping program on hip and lumbar spine bone mineral content (BMC) in prepubescent children
Sadres et al. [56]	2001	24	-	9.20 ± 0.30	Not Defined	To examine the effect of two school years of progressive resistance training (9 months of training, 3 months of detraining, and 9 months of training) on muscle strength, linear growth, and self-concept as well as to monitor the injury rate during this program among prepubertal boys
Faigenbaum et al. [61]	2002	26	16	9.95 ± 1.40	Tier 1—RcCh Recreational Physical Activity	To compare the responses of a 1 and 2 day per week strength training program on upper body strength, lower body strength, and motor performance ability in children
Faigenbaum et al. [62]	2005	17	14	10.40 ± 1.20	Tier 1—RcCh Recreational Physical Activity	To compare the effects of a low RM (6–10 RM) and a high RM (15–20 RM) resistance training program on measures of muscular fitness in untrained children
Ingle et al. [63]	2006	33	-	12.30 ± 0.30	Tier 1—RcCh Recreational Physical Activity	To determine the effect of an upper and lower body complex training and detraining program in pre- and early pubertal boys
Faigenbaum et al. [64]	2009	23	17	9.00 ± 0.90	Tier 0—SdCh Sedentary Lifestyle	To examine the effects of plyometric training on the fitness performance of elementary school physical education students
Granacher et al. [65]	2011	8	9	8.60 ± 0.50	Tier 1—RcCh Recreational Physical Activity	To investigate the effects of standardized high-intensity strength training (HIS) on knee extensor/flexor strength, countermovement jump (CMJ) height, static postural control, soft lean mass, and cross-sectional area (CSA) of the quadriceps muscle of the dominant leg
Souissi et al. [66]	2012	16	-	10.50 ± 0.50	Tier 0—SdCh Sedentary Lifestyle	To investigate the effect of 6 weeks of resistance training scheduled in the morning or evening hours on the daily variations of muscle strength and power during short-lasting physical tests in 10–11-year-old boys
Michailidis et al. [67]	2013	24	-	10.60 ± 0.60	Tier 2—TrCh Regularly Training	To investigate whether the combination of soccer practice and plyometric training (PT) would enhance athletic ability and soccer-specific performance to a greater extent than soccer practice alone in prepubertal soccer players
Ramírez-Campillo et al. [68]	2014	37	-	10.40 ± 2.30	Tier 2—TrCh Regularly Training	To compare the effects of plyometric training using 30, 60, or 120 s of rest between sets on explosive adaptations in young soccer players
Waugh et al. [69]	2014	4	5	8.90 ± 0.20	Tier 0—SdCh Sedentary Lifestyle	To examine the effects of plantar flexor resistance training (RT) on the mechanical properties of the Achilles tendon in prepubertal children and to determine the mechanisms underpinning potential adaptations
Cunha et al. [70]	2015	9	-	10.40 ± 0.50	Tier 1—RcCh Recreational Physical Activity	To investigate the effects of resistance training (RT) on the neuromuscular and cardiorespiratory performance, body composition, and bone mineral content (BMC) of healthy prepubertal boys
Rodríguez-Rosell et al. [71]	2016	15	-	12.70 ± 0.50	Tier 2—TrCh Regularly Training	To examine the effects of 6 weeks of resistance training (RT) with low loads (~45–60% 1 RM) and low volume (2 or 3 sets and 4–8 repetitions/set) combined with jumps and sprints on lower limb muscle strength, jumping ability, and acceleration capacity in pre-peak high velocity (PHV) soccer players
Negra et al. [72]	2018	13	-	12.70 ± 0.20	Tier 2—TrCh Regularly Training	To examine the effects of an 8-week plyometric jump training (PJT) program on changes of direction (CoD), speed, jump performance, and repeated-sprint ability (RSA) in prepubertal male soccer players
Drouzas et al. [73]	2020	46	-	9.95 ± 1.15	Tier 2—TrCh Regularly Training	To evaluate the effects of 10 weeks of periodized unilateral and bilateral plyometric training on strength, sprint, and jumping performance in preadolescent soccer athletes
Almeida et al. [74]	2021	64	-	7.90 ± 0.90	Tier 0—SdCh Sedentary Lifestyle	To examine the effects of plyometric training (12 weeks, twice/week, 20 min/day) on physical fitness and gross motor coordination in schoolboys aged 7–9 years
Padrón-Cabo et al. [75]	2021	10	-	12.60 ± 0.70	Tier 2—TrCh Regularly Training	To examine the effects and determine whether plyometric drills with an agility ladder are an effective training strategy to develop jumping, sprinting, and agility performance in prepubertal players
Sammoud et al. [76]	2021	-	12	10.01 ± 0.57	Tier 2—TrCh Regularly Training	To study the effects of an 8-week plyometric jump training (PJT) program in combination with regular swimming training compared with swimming training alone on measures of muscle power and sport-specific performances in prepubertal female swimmers
Wick et al. [77]	2021	16	16	4.60 ± 0.80	Tier 0—SdCh Sedentary Lifestyle	To examine the effects of an integrative strength-dominated exercise program on measures of physical fitness and cognitive performance in preschool children

Note: M, male; F, female; “-”, not defined. Physical activity level according to classification by McKay et al. [44]: tier 0 or sedentary children—“SdCh”, occasional and/or incidental physical activity; tier 1 or recreationally active children—“RcCh”, may participate in multiple sports/forms of activity; tier 2 or trained children—“TrCh”, training with a purpose to compete in a specific sport or discipline.

**Table 3:** Strength training programs (STPs) registered according to the strength training variables (STVs), expressed by absolute (*n*) and relative (%) frequency.

STV	STP—by Strength Training Variable				
Periodization	Undulating		Linear/Non-Linear		No Periodization
	14 (13.46%)		87 (83.65%)		3 (2.89%)
Duration	<8 weeks		8–12 weeks		>12 weeks
	9 (8.65%)		85 (81.73%)		10 (9.62%)
Frequency	1 day		2 days		3 days
	4 (3.85%)		85 (81.73%)		15 (14.42%)
Intensity	Low		Moderate		High
	16 (15.69%)		63 (61.76%)		23 (22.55%)
Volume	Low		Moderate		High
	92 (88.46%)		6 (5.77%)		6 (5.77%)
Movement Velocity	Low		Moderate		High
	2 (5.71%)		4 (11.43%)		29 (82.86%)
Rest Interval	Short		Medium		Long
	4 (8.16%)		25 (51.02%)		20 (40.82%)
Joints Involved	Single-joint			Multi-joint	
	29 (27.88%)			75 (72.12%)	
Exercise Type	Ply	BE	F-WE	M-BE	WE
	24 (23.08%)	27 (25.95%)	11 (10.58%)	38 (36.54%)	4 (3.85%)

Notes: Ply, plyometrics; BE, bodyweight exercise; F-WE, free weight exercise; M-BE, machine-based exercises; WE, weightlifting exercise. Data are presented according to the total number of different STPs implemented (*n* = 104), except for intensity (*n* = 102), movement velocity (*n* = 35), and rest interval (*n* = 49).

**Table 4:** Main neuromuscular (NAs) and morphological (MAs) adaptations (*n*) from strength training in prepubertal children (based on the number of strength training programs (STP) implemented) according to the strength training variables (STVs).

STV	STP	NA—GSS		NA—BPA					MA—BC	
		Jump	Sprint	Strength	Agility	Coordination	Balance	Flexibility	BF	LBM
		↑	↑	↑	↑	↑	↑	↑	↓	↑
Periodization	Undulating	14 *	7 */7 ^†^	8 */4 ^†^	2 */2 ^†^	-	6 ^†^	-	6	6
	Linear	12 */20 ^†^	6 */1 ^†^	55 */24 ^†^	-	2 *	-	4 */16 ^†^	13	11
	No periodization	3 *	5 */3 ^†^	3 *	3 *	-	-	-	-	-
Duration	<8 weeks	9 *	3 */2 ^†^	8 *	4 *	-	-	-	2	2
	8–12 weeks	20 */20 ^†^	10 */6 ^†^	50 */28 ^†^	1 */2 ^†^	2 *	6 ^†^	4 */16 ^†^	17	15
	>12 weeks	-	-	8 *	-	-	-	-	-	-
Frequency	1 day	4 ^†^	-	4 ^†^	-	-	-	4 ^†^	-	-
	2 days	18 */16 ^†^	7 */7 ^†^	60 */20 ^†^	5 */2 ^†^	2 *	2 ^†^	4 */12 ^†^	12	10
	3 days	11 *	6 */1 ^†^	6 */4 ^†^	-	-	4 ^†^	-	7	7
Intensity	Low	12 *	5 */3 ^†^	13 */2 ^†^	1 *	2 *	2 ^†^	2 ^†^	5	4
	Moderate	6 */16 ^†^	5 */1 ^†^	45 */12 ^†^	1 *	-	-	4 */8 ^†^	13	12
	High	9 */4 ^†^	3 */4 ^†^	8 */12 ^†^	3 */2 ^†^	-	2 ^†^	6 ^†^	1	1
Volume	Low	25 */19 ^†^	11 */6 ^†^	55 */28 ^†^	5 */2 ^†^	1 *	6 ^†^	4 */14 ^†^	12	10
	Moderate	2 *	2 *	6 *	-	-	-	-	6	6
	High	2 */1 ^†^	2 ^†^	5 *	-	1 *	-	2 ^†^	1	1
Movement Velocity	Low	-	11 */7 ^†^	4 *	-	-	-	-	-	-
	Moderate	4 ^†^	-	18 *	-	-	-	-	-	-
	High	20 *	-	22 */8 ^†^	5 */2 ^†^	2 *	2 ^†^	4 ^†^	6	4
Rest Interval	Short	4 *	2 */2 ^†^	3 *	2 *	-	-	-	2	2
	Medium	6 */8 ^†^	5 */3 ^†^	17 */4 ^†^	2 *	2 *	-	10 ^†^	10	10
	Long	8 */4 ^†^	7 */2 ^†^	20 *	1 */2 ^†^	-	2 ^†^	-	2	-
Joints Involved	Single-joint	4 */2 ^†^	3 *	18 */8 ^†^	-	-	-	2 ^†^	6	6
	Multi-joint	25 */18 ^†^	10 */8 ^†^	48 */20 ^†^	5 */2 ^†^	2 *	6 ^†^	4 */14 ^†^	13	11
Exercise Type	Plyometrics	21 *	5 */9 ^†^	16 */2 ^†^	5 */2 ^†^	2 *	4 ^†^	4 ^†^	7	5
	Bodyweight	3 */8 ^†^	3 */2 ^†^	13 */14 ^†^	-	-	2 ^†^	2 */6 ^†^	-	-
	Free weight	3 *	10 */6 ^†^	11 *	-	-	-	-	6	6
	Machine-based	2 */12 ^†^	-	22 */12 ^†^	-	-	-	2 */6 ^†^	6	6
	Weightlifting	-	-	4 *	-	-	-	-	-	-

Notes: NA-GSS, neuromuscular adaptations—general sports skills; NA-BPA, neuromuscular adaptations—basic physical abilities; MA-BC, morphological adaptations—body composition; BF, body fat; LBM, lean body mass; ↑, increase; ↓, decrease; †, no significant increase/decrease; *, significant increase/decrease.

**Table 5:** External and internal quality assessment—PEDro scale.

Study/Item	1	2	3	4	5	6	7	8	9	10	11	Total
Tsolakis et al. [57]	0	1	0	1	0	0	0	1	1	1	1	6
Diallo et al. [58]	0	1	0	1	0	0	0	1	1	1	1	6
Faigenbaum et al. [59]	1	1	1	1	0	0	0	1	1	1	1	7
Fuchs et al. [60]	1	1	0	1	0	0	0	1	1	1	1	6
Sadres et al. [56]	1	1	1	1	0	0	0	1	1	1	1	7
Faigenbaum et al. [61]	1	1	0	1	0	0	0	1	1	1	1	6
Faigenbaum et al. [62]	1	1	1	1	0	0	0	1	1	1	1	7
Ingle et al. [63]	1	1	0	1	0	0	0	1	1	1	1	6
Faigenbaum et al. [64]	1	1	0	1	0	0	0	1	1	1	1	6
Granacher et al. [65]	0	1	0	1	0	0	0	1	1	1	1	6
Souissi et al. [66]	0	1	0	1	0	0	0	1	1	1	1	6
Michailidis et al. [67]	1	1	0	1	0	0	0	1	1	1	1	6
Ramírez-Campillo et al. [68]	1	1	0	1	0	0	0	1	1	1	1	6
Waugh et al. [69]	0	1	0	1	0	0	0	1	1	1	1	6
Cunha et al. [70]	0	1	0	1	0	0	0	1	1	1	1	6
Rodríguez-Rosell et al. [71]	1	1	0	1	0	0	0	1	1	1	1	6
Negra et al. [72]	0	1	0	1	0	0	0	1	1	1	1	6
Drouzas et al. [73]	1	1	0	1	0	0	0	1	1	1	1	6
Almeida et al. [74]	1	1	0	1	0	0	0	1	1	1	1	6
Sammoud et al. [76]	0	1	0	1	0	0	0	1	1	1	1	6
Padrón-Cabo et al. [75]	1	1	1	0	0	0	0	1	1	1	1	6
Wick et al. [77]	1	1	0	0	0	0	0	0	1	1	1	4

Notes: 1 = eligibility criteria were specified; 2 = subjects were randomly allocated to groups (in a crossover study, subjects were randomly allocated an order in which treatments were received); 3 = allocation was concealed; 4 = the groups were similar at baseline regarding the most important prognostic indicators; 5 = there was blinding of all subjects; 6 = there was blinding of all therapists who administered the therapy; 7 = there was blinding of all assessors who measured at least one key outcome; 8 = measures of at least one key outcome were obtained from more than 85% of the subjects initially allocated to groups; 9 = all subjects for whom outcome measures were available received the treatment or control condition as allocated or, where this was not the case, data for at least one key outcome were analyzed by “intention to treat”; 10 = the results of between-group statistical comparisons are reported for at least one key outcome; 11 = the study provides both point measures and measures of variability for at least one key outcome.

## Data Availability

The raw data supporting the conclusions of this article will be made available by the authors without undue reservation.

## References

[1] Moritani T., de Vries H.A. (1979). Neural factors versus hypertrophy in the time course of muscle strength gain. Am. J. Phys. Med..

[2] Stone M.H. (1993). Position Statement: Explosive Exercise and Training. Natl. Strength Cond. Assoc. J..

[3] Möck S., Hartmann R., Wirth K., Rosenkranz G., Mickel C. (2021). Relationship Between Maximal Dynamic Force in the Deep Back Squat and Sprinting Performance in Consecutive Segments Up to 30 m. J. Strength Cond. Res..

[4] Maestroni L., Read P., Bishop C., Papadopoulos K., Suchomel T.J., Comfort P., Turner A. (2020). The Benefits of Strength Training on Musculoskeletal System Health: Practical Applications for Interdisciplinary Care. Sport. Med..

[5] Gabbett T.J. (2018). Debunking the myths about training load, injury and performance: Empirical evidence, hot topics and recommendations for practitioners. Br. J. Sport. Med..

[6] Coratella G. (2022). Appropriate Reporting of Exercise Variables in Resistance Training Protocols: Much more than Load and Number of Repetitions. Sport. Med. Open.

[7] González-Badillo J.J., Ribas Serna J. (2019). Fuerza, Velocidad y Rendimiento Físico y Deportivo.

[8] Rexach J.A.S., Ruiz J.R., Bustamante-Ara N., Villarán M.H., Gil P.G., Sanz Ibáñez M.J., Sanz N.B., Santamaría V.O., Sanz N.G., Prada A.B.M. (2009). Health enhancing strength training in nonagenarians (STRONG): Rationale, design and methods. BMC Public Health.

[9] Fragala M.S., Cadore E.L., Dorgo S., Izquierdo M., Kraemer W.J., Peterson M.D., Ryan E.D. (2019). Resistance Training for Older Adults. J. Strength Cond. Res..

[10] Hagstrom A.D., Marshall P.W.M., Lonsdale C., Cheema B.S., Fiatarone Singh M.A., Green S. (2016). Resistance training improves fatigue and quality of life in previously sedentary breast cancer survivors: A randomised controlled trial. Eur. J. Cancer Care.

[11] da Rocha P.E.C.P., da Silva V.S., Camacho L.A.B., Vasconcelos A.G.G. (2015). Efeitos de longo prazo do treinamento resistido nos indicadores de obesidade: Uma revisão sistemática. Rev. Bras. Cineantropometria E Desempenho Hum..

[12] Perales M., Santos-Lozano A., Ruiz J.R., Lucia A., Barakat R. (2016). Benefits of aerobic or resistance training during pregnancy on maternal health and perinatal outcomes: A systematic review. Early Hum. Dev..

[13] Myer G., Faigenbaum A., Chu D., Falkel J., Ford K., Best T., Hewett T. (2011). Integrative Training for Children and Adolescents: Techniques and Practices for Reducing Sports-Related Injuries and Enhancing Athletic Performance. Phys. Sport. Med..

[14] Collins H., Booth J.N., Duncan A., Fawkner S. (2019). The effect of resistance training interventions on fundamental movement skills in youth: A meta-analysis. Sport. Med. Open.

[15] Roberts C. (2016). Tanner’s Puberty Scale: Exploring the historical entanglements of children, scientific photography and sex. Sexualities.

[16] Vrijens J. (1978). Muscle strength development in the pre-and post-pubescent age. Pediatr. Work Physiol..

[17] Docherty D. (1987). The effects of variable speed resistance training on strength development in prepubertal boys. J. Hum. Mov. Stud..

[18] American Academy of Pediatrics Committee on Sports Medicine (1990). Strength Training, Weight and Power Lifting, and Body Building by Children and Adolescents. Pediatrics.

[19] Faigenbaum A.D., Kraemer W.J., Blimkie C.J.R., Jeffreys I., Micheli L.J., Nitka M., Rowland T.W. (2009). Youth Resistance Training: Updated Position Statement Paper From the National Strength and Conditioning Association. J. Strength Cond. Res..

[20] Lloyd R.S., Faigenbaum A.D., Stone M.H., Oliver J., Jeffreys I., Moody J.A., Brewer C., Pierce K.C., McCambridge T.M., Howard R. (2014). Position statement on youth resistance training: The 2014 International Consensus. Br. J. Sport. Med..

[21] Lloyd R.S., Cronin J.B., Faigenbaum A.D., Haff G.G., Howard R., Kraemer W.J., Micheli L.J., Myer G.D., Oliver J. (2016). National Strength and Conditioning Association Position Statement on Long-Term Athletic Development. J. Strength Cond. Res..

[22] Faigenbaum A.D., Micheli L.J. (2017). Youth Strenght Training.

[23] World Health Organization (2022). Physical Activity.

[24] Rogol A.D., Clark P.A., Roemmich J.N. (2000). Growth and pubertal development in children and adolescents: Effects of diet and physical activity. Am. J. Clin. Nutr..

[25] Pitton P.M. (1992). Prepubescent Strength Training: The Effects of Resistance Training on Strength Gains In Prepubescent Children. Natl. Strength Cond. Assoc. J..

[26] Shanmugam C., Maffulli N. (2008). Sports injuries in children. Br. Med. Bull..

[27] Behm D.G., Faigenbaum A.D., Falk B., Klentrou P. (2008). Canadian Society for Exercise Physiology position paper: Resistance training in children and adolescents. Appl. Physiol. Nutr. Metab..

[28] Valovich McLeod T.C., Decoster L.C., Loud K.J., Micheli L.J., Parker J.T., Sandrey M.A., White C. (2011). National Athletic Trainers’ Association Position Statement: Prevention of Pediatric Overuse Injuries. J. Athl. Train..

[29] Alentorn-Geli E., Myer G.D., Silvers H.J., Samitier G., Romero D., Lázaro-Haro C., Cugat R. (2009). Prevention of non-contact anterior cruciate ligament injuries in soccer players. Part 2: A review of prevention programs aimed to modify risk factors and to reduce injury rates. Knee Surg. Sport. Traumatol. Arthrosc..

[30] Bahr R., Thorborg K., Ekstrand J. (2015). Evidence-based hamstring injury prevention is not adopted by the majority of Champions League or Norwegian Premier League football teams: The Nordic Hamstring survey. Br. J. Sport. Med..

[31] McCall A., Carling C., Nedelec M., Davison M., Le Gall F., Berthoin S., Dupont G. (2014). Risk factors, testing and preventative strategies for non-contact injuries in professional football: Current perceptions and practices of 44 teams from various premier leagues. Br. J. Sport. Med..

[32] Folland J.P., Williams A.G. (2007). The Adaptations to Strength Training. Sport. Med..

[33] Cormie P., McGuigan M.R., Newton R.U. (2011). Developing Maximal Neuromuscular Power. Part 2—Training Considerations for Improving Maximal Power Production. Sport. Med..

[34] Behringer M., Heede A.V., Matthews M., Mester J. (2011). Effects of strength training on motor performance skills in children and adolescents: A meta-analysis. Pediatr. Exerc. Sci..

[35] Ozmun J.C., Mikesky A.E., Surburg P.R. (1994). Neuromuscular adaptations following prepubescent strength training. Med. Sci. Sport. Exerc..

[36] Cormie P., McGuigan M.R., Newton R.U. (2011). Developing Maximal Neuromuscular Power. Sport. Med..

[37] Hong A.R., Kim S.W. (2018). Effects of Resistance Exercise on Bone Health. Endocrinol. Metab..

[38] Lazarczuk S.L., Maniar N., Opar D.A., Duhig S.J., Shield A., Barrett R.S., Bourne M.N. (2022). Mechanical, Material and Morphological Adaptations of Healthy Lower Limb Tendons to Mechanical Loading: A Systematic Review and Meta-Analysis. Sport. Med..

[39] Peña G., Heredia J.R., Lloret C., Martín M., Da Silva-Grigoletto M.E. (2016). Introduction to strength training at early age: A review. Rev. Andal. Med. Del Deport..

[40] Faigenbaum A.D., Myer G.D. (2010). Resistance training among young athletes: Safety, efficacy and injury prevention effects. Br. J. Sport. Med..

[41] Moher D., Shamseer L., Clarke M., Ghersi D., Liberati A., Petticrew M., Shekelle P., Stewart L.A. (2015). Preferred reporting items for systematic review and meta-analysis protocols (PRISMA-P) 2015 statement. Syst. Rev..

[42] Liberati A., Altman D.G., Tetzlaff J., Mulrow C., Gøtzsche P.C., Ioannidis J.P.A., Clarke M., Devereaux P.J., Kleijnen J., Moher D. (2009). The PRISMA statement for reporting systematic reviews and meta-analyses of studies that evaluate health care interventions: Explanation and elaboration. PLoS Med..

[43] Baxter-Jones A.D.G., Eisenmann J.C., Sherar L.B. (2005). Controlling for maturation in pediatric exercise science. Pediatr. Exerc. Sci..

[44] McKay A.K.A., Stellingwerff T., Smith E.S., Martin D.T., Mujika I., Goosey-Tolfrey V.L., Sheppard J., Burke L.M. (2022). Defining Training and Performance Caliber: A Participant Classification Framework. Int. J. Sport. Physiol. Perform..

[45] Williams T.D., Tolusso D.V., Fedewa M.V., Esco M.R. (2017). Comparison of Periodized and Non-Periodized Resistance Training on Maximal Strength: A Meta-Analysis. Sport. Med..

[46] Clark J.E. (2016). The impact of duration on effectiveness of exercise, the implication for periodization of training and goal setting for individuals who are overfat, a meta-analysis. Biol. Sport.

[47] Lesinski M., Prieske O., Granacher U. (2016). Effects and dose-response relationships of resistance training on physical performance in youth athletes: A systematic review and meta-analysis. Br. J. Sport. Med..

[48] Grgic J., Schoenfeld B.J., Davies T.B., Lazinica B., Krieger J.W., Pedisic Z. (2018). Effect of Resistance Training Frequency on Gains in Muscular Strength: A Systematic Review and Meta-Analysis. Sport. Med..

[49] Williams P.E. (2007). Practical Guidelines for Plyometric Intensity. Sport. Med..

[50] Fort-Vanmeerhaeghe A., Romero-Rodriguez D., Lloyd R.S., Kushner A., Myer G.D. (2016). Integrative Neuromuscular Training in Youth Athletes. Part II: Strategies to Prevent Injuries and Improve Performance. Strength Cond. J..

[51] Ralston G.W., Kilgore L., Wyatt F.B., Baker J.S. (2017). The Effect of Weekly Set Volume on Strength Gain: A Meta-Analysis. Sport. Med..

[52] Grgic J., Schoenfeld B.J., Skrepnik M., Davies T.B., Mikulic P. (2018). Effects of Rest Interval Duration in Resistance Training on Measures of Muscular Strength: A Systematic Review. Sport. Med..

[53] Suchomel T.J., Nimphius S., Bellon C.R., Stone M.H. (2018). The Importance of Muscular Strength: Training Considerations. Sport. Med..

[54] Maher C.G., Sherrington C., Herbert R.D., Moseley A.M., Elkins M. (2003). Reliability of the PEDro scale for rating quality of randomized controlled trials. Phys. Ther..

[55] Tanner J.M. (1962). Growth at Adolescence.

[56] Sadres E., Eliakim A., Constantini N., Lidor R., Falk B. (2001). The Effect of Long-Term Resistance Training on Anthropometric Measures, Muscle Strength, and Self Concept in Pre-Pubertal Boys. Pediatr. Exerc. Sci..

[57] Tsolakis C., Messinis D., Stergioulas A., Dessypris A. (2000). Hormonal Responses after Strength Training and Detraining in Prepubertal and Pubertal Boys. J. Strength Cond. Res..

[58] Diallo O., Dore E., Duche P., Van Praagh E. (2001). Effects of plyometric training followed by a reduced training programme on physical performance in prepubescent soccer players. J. Sport. Med. Phys. Fit..

[59] Faigenbaum A.D., Loud R.L., OʼConnel J., Glover S., OʼConnel J., Wescott W.L. (2001). Effects of Different Resistance Training Protocols on Upper-Body Strength and Endurance Development in Children. J. Strength Cond. Res..

[60] Fuchs R.K., Bauer J.J., Snow C.M. (2001). Jumping Improves Hip and Lumbar Spine Bone Mass in Prepubescent Children: A Randomized Controlled Trial. J. Bone Miner. Res..

[61] Faigenbaum A.D., Milliken L.A., Loud R.L., Burak B.T., Doherty C.L., Wescott W.L. (2002). Comparison of 1 and 2 Days per Week of Strength Training in Children. Res. Q. Exerc. Sport.

[62] Faigenbaum A.D., Milliken L., Moulton L., Westcott W.L. (2005). Early Muscular Fitness Adaptations in Children in Response to Two Different Resistance Training Regimens. Pediatr. Exerc. Sci..

[63] Ingle L., Sleap M., Tolfrey K. (2006). The effect of a complex training and detraining programme on selected strength and power variables in early pubertal boys. J. Sport. Sci..

[64] Faigenbaum A.D., Farrell A.C., Radler T., Zbojovsky D., Chu D.A., Ratamess N.A., Kang J.H., Hoffman J.R. (2009). ‘Plyo Play’: A Novel Program of Short Bouts of Moderate and High Exercise Improves Physical Fitness in Elementary School Chlidren. Phys. Educ..

[65] Granacher U., Goesele A., Roggo K., Wischer T., Fischer S., Zuerny C., Gollhofer A., Kriemler S. (2011). Effects and Mechanisms of Strength Training in Children. Int. J. Sport. Med..

[66] Souissi H., Chtourou H., Chaouachi A., Dogui M., Chamari K., Souissi N., Amri M. (2012). The Effect of Training at a Specific Time-of-Day on the Diurnal Variations of Short-Term Exercise Performances in 10- to 11-Year-Old Boys. Pediatr. Exerc. Sci..

[67] Michailidis Y., Fatouros I.G., Primpa E., Michailidis C., Avloniti A., Chatzinikolau A., Barbero-Alvarez J.C., Tsoukas D., Douroudos I.I., Draganidis D. (2013). Plyometrics’ Trainability in Preadolescent Soccer Athletes. J. Strength Cond. Res..

[68] Ramírez-Campillo R., Andrade D.C., Álvarez C., Henríquez-Olguín C., Martínez C., Báez-Sanmartín E., Silva-Urra J., Burgos C., Izquierdo M. (2014). The Effects of Interset Rest on Adaptation to 7 Weeks of Explosive Training in Young Soccer Players. J. Sport. Sci. Med..

[69] Waugh C.M., Korff T., Fath F., Blazevich A.J. (2014). Effects of resistance training on tendon mechanical properties and rapid force production in prepubertal childre. J. Appl. Physiol..

[70] Cunha G., Morganti M., Cadore E., de Oliveira N.L., Bos dos Santos C., Silveira Pinto R., Reischak-Olivera A. (2015). Physiological Adaptations to Resistance Training in Prepubertal Boys. Res. Q. Exerc. Sport.

[71] Rodríguez-Rosell D., Franco-Marquez F., Pareja-Blanco F., Mora-Custodio R., Yañez-García J.M., Gonzalez-Suárez J.M., González-Badillo J. (2016). Effects of 6 Weeks Resistance Training Combined With Plyometric and Speed Exercises on Physical Performance of Pre-Peak-Height-Velocity Soccer Players. Int. J. Sport. Physiol. Perform..

[72] Negra Y., Chaabene H., Fernández-Fernández J., Sammoud S., Bouguezzi R., Prieske O., Granacher U. (2018). Short-term plyometric jump training improves repeated-sprint ability in prepuberal male soccer players. J. Strength Cond. Res..

[73] Drouzas V., Katsikas C., Zafeiridis A., Jamurtas A.Z., Bogdanis G.C. (2020). Unilateral Plyometric Training is Superior to Volume-Matched Bilateral Training for Improving Strength, Speed and Power of Lower Limbs in Preadolescent Soccer Athletes. J. Hum. Kinet..

[74] Almeida M., Leandro C.G., Queiroz D.R., José-da-silva M., Pessoa dos Prazeres T.M., Pereira G.M., Silva das-Neves G., Carneiro R.C., Figueredo-Alves A.D., Nakamura F.Y. (2021). Plyometric training increases gross motor coordination and associated components of physical fitness in children. Eur. J. Sport Sci..

[75] Padrón-Cabo A., Lorenzo-Martínez M., Perez-Ferreiros A., Costa P.B., Rey E. (2021). Effects of Plyometric Training with Agility Ladder on Physical Fitness in Youth Soccer Players. Int. J. Sport. Med..

[76] Sammoud S., Negra Y., Bouguezzi R., Hachana Y., Granacher U. (2021). The effects of plyometric jump training on jump and sport-speci fi c performances in prepubertal female swimmers. J. Exerc. Sci. Fit..

[77] Wick K., Kriemler S., Granacher U. (2021). Effects of a Strength-Dominated Exercise Program on Physical Fitness and Cognitive Performance in Preschool Children. J. Strength Cond. Res..

[78] Sgro M., McGuigan M.R., Pettigrew S., Newton R.U. (2009). The Effect of Duration of Resistance Training Interventions in Children Who Are Overweight or Obese. J. Strength Cond. Res..

[79] Faigenbaum A.D., McFarland J.E., Johnson L., Kang J.H., Bloom J., Ratamess N.A., Hoffman J.R. (2007). Preliminary Evaluation of an After-School Resistance Training Program for Improving Physical Fitness in Middle School-Age Boys. Percept. Mot. Skills.

[80] Faigenbaum A.D., McFarland J.E. (2016). Resistance training for kids: Right from the Start. ACSM’s Health Fit. J..

[81] Vingren J., Kraemer W.J., Ratamess N.A., Anderson J.M., Volek J.S., Maresh C.M. (2010). Testosterone Physiology in Resistance Exercise and Training. Sport. Med..

[82] de Villarreal E.S.S., González-Badillo J.J., Izquierdo M. (2008). Low and Moderate Plyometric Training Frequency Produces Greater Jumping and Sprinting Gains Compared with High Frequency. J. Strength Cond. Res..

[83] Schonfeld B.J., Contreras B., Krieger J., Grgic J., del Castillo K., Belliard R., Alto A. (2019). Resistance Training Volume Enhances Muscle Hypertrophy but Not Strength in Trained Men. Med. Sci. Sport. Exerc..

[84] González-Badillo J.J., Gorostiaga E.M., Arellano R., Izquierdo M. (2005). Moderate Resistance Training Volume Produces More Favorable Strength Gains Than High or Low Volumes During a Short-Term Training Cycle. J. Strength Cond. Res..

[85] Baena-Marín M., Rojas-Jaramillo A., González-Santamaría J., Rodríguez-Rosell D., Petro J., Kreider R.B., Bonilla D.A. (2022). Velocity-Based Resistance Training on 1-RM, Jump and Sprint Performance: A Systematic Review of Clinical Trials. Sports.

[86] Casolo A., Farina D., Falla D., Bazzucchi I., Felici F., del Vecchio A. (2020). Strength Training Increases Conduction Velocity of High-Threshold Motor Units. Med. Sci. Sport. Exerc..

[87] Pareja-Blanco F., Rodríguez-Rosell D., Sánchez-Medina L., Gorostiaga E., González-Badillo J. (2014). Effect of Movement Velocity during Resistance Training on Neuromuscular Performance. Int. J. Sport. Med..

[88] Schoenfeld B.J., Pope Z.K., Benik F.M., Hester G.M., Sellers J., Nooner J., Schnaiter J.A., Bond-Williams K.E., Carter A.S., Ross C.L. (2016). Longer Interset Rest Periods Enhance Muscle Strength and Hypertrophy in Resistance-Trained Men. J. Strength Cond. Res..

[89] Falk B., Dotan R. (2006). Child-Adult Differences in the Recovery from High-Intensity Exercise. Exerc. Sport Sci. Rev..

[90] Brearley S., Bishop C. (2019). Transfer of Training: How Specific Should We Be?. Strength Cond. J..

[91] Fouré A., Nordez A., Cornu C. (2010). Plyometric training effects on Achilles tendon stiffness and dissipative properties. J. Appl. Physiol..

[92] Manna I. (2014). Growth Development and Maturity in Children and Adolescent: Relation to Sports and Physical Activity. Am. J. Sport. Sci. Med..

[93] Stratton M.T., Tinsley G.M., Alesi M.G., Hester G.M., Olmos A.A., Serafini P.R., Modjeski A.S., Mangine G.T., King K., Savage S.N. (2020). Four Weeks of Time-Restricted Feeding Combined with Resistance Training Does Not Differentially Influence Measures of Body Composition, Muscle Performance, Resting Energy Expenditure, and Blood Biomarkers. Nutrients.

[94] Westcott W.L. (2012). Resistance Training is Medicine. Curr. Sport. Med. Rep..

[95] McCarthy H.D., Cole T.J., Fry T., Jebb S.A., Prentice A.M. (2006). Body fat reference curves for children. Int. J. Obes..

[96] Marques-Vidal P., Marcelino G., Ravasco P., Camilo M.E., Oliveira J.M. (2008). Body fat levels in children and adolescents: Effects on the prevalence of obesity. E. Spen. Eur. E. J. Clin. Nutr. Metab..

